# A novel protein biochip screening serum anti-sperm antibody expression and natural pregnancy rate in a follow-up study in Chinese infertility

**DOI:** 10.1042/BSR20191769

**Published:** 2020-02-11

**Authors:** Feihong Xu, Lei Ye, Yuan Hu, Chengyun Cai, Zhen Wang, Liqing Fan, Lihua Song, Zhenshan Xu, Weidong Du

**Affiliations:** 1 Department of Medicine, Immunology Institute, Icahn School of Medicine at Mount Sinai, New York, NY 10029, U.S.A.; 2 Department of Neurosurgery, First Affiliated Hospital of Anhui Medical University, Hefei 230022, Anhui, P.R. China; 3 Anhui Academic Institute of Biology, Haiguan Road K-1, Hefei 230088, Anhui, P.R. China; 4 Reproductive and Genetic Hospital of CITIC Xiangya, Hunan 410008, P.R. China; 5 Department of Pathology, Anhui Medical University, Hefei 230032, Anhui, P.R. China

**Keywords:** anti-sperm antibody, immunological assay, infertility, natural pregnancy rate, protein biochip

## Abstract

Production of anti-sperm antibody (ASA) often suffers from autoimmune reaction against sperms in human infertility. The antibodies are measured in both blood and seminal plasma of males. Here, we reported a simple protein biochip methodology that takes advantage of a functionalized self-assembled monolayer modified by N-hydroxysuccinimide (NHS) and enables identification of anti-sperm antibody in Chinese male infertility. To validate this biochip platform, we immobilized purified sperm protein on the biochip surface and tested a variety of parameters in quality controls for the protein assay, respectively. Then, we analyzed serum samples from 368 patients with infertility and 116 healthy donors by means of this biochip simultaneously. We found that positive rate of serum ASA was 20.92% (77/368) in the cases and 1.72% (2/116) in the controls, respectively. Furthermore, we further corroborated the biochip assay in comparison with ELISA method. We found that both methods were compatible for the detection of serum ASA in the patients. In addition, a follow-up study for natural conception in ASA-positive and ASA-negative patients was conducted. The result showed a significant correlation between serum ASA expression and natural pregnancy rate 6.5% in ASA-positive patients while 18.9% in ASA-negative patients, indicating the potential roles of ASA in naturally reproductive processes.

## Introduction

Decreased reproductive rate in human being has become a critical social problem worldwide over the last decades. Approximately, 50% of the total couple’s inability to bear children was attributed to male infertility, either solely or in combination with female factors [1]. It is noteworthy that quality and quantity of human sperms has kept declining since 1940s [2–5].

Multi-factorial disorders, including genetic abnormalities and/or environment influences, potentially affect fertility parameters. It is demonstrated that abnormalities of nuclear DNA [6], mitochondrial DNA [7] and RNA [8] in human being might either directly or indirectly affect the spermatogenesis. The impact of exposure to environmental factors would be indirect but significant. It is reported that male reproductive system is extremely sensitive toward the environmental exposures, including radiation, toxicants, stress, injury of testis and autoimmune turbulence [9–11].

Among the environmental exposures, anti-sperm antibody (ASA), which suffers from autoimmune reaction of human, has been aroused more attention to the early stage of human infertility. ASA is detected, once produced, in both blood and seminal plasma of males [12]. The origin of ASA in fertile male reproductive tract is yet unknown. However, damage of the blood–testis barrier acting to separate haploid cells, sperms, and its precursors from the human immune system may obviously play a pivotal role in the production of ASA. It is well known that varicocele, congenital absence of vasdeferens and vasectomy or trauma would make sperms exposed to the immune system and be susceptible to inducing production of ASA at higher risk [13,14]. It is reported that approximately 50% male produce ASA after vasectomy [15]. Reactive oxygen species (ROS) production in ASA-positive patients was 2.8 and 3.5 times higher than those of ASA-negative patients and fertile man, respectively [14]. Currently, the detection of ASA is widely carried out in laboratories and hospitals in China. Commercially available kits of enzyme-linked immunosorbent assay (ELISA), mixed anti-globulin reaction test (MAR) or immune beads test (IBT) are the most useful approaches in screening serum ASA, though results from the methodologies often vary somehow.

Protein microarray is an innovative technology and has been widely applied in numerous proteomic-based studies [16]. By integrating targeted proteins on a solid substrate, researchers could simultaneously interrogate the interaction and function of proteins or biomolecules at high density [17]. In comparison with other conventional approaches for protein detection, protein biochip provided extremely high sensitively and specificity. Furthermore, the high-throughput property and minimal input of sample also reveal its potential clinical impressive superiority [18,19].

In the present study, we proposed a biochip-based approach for fast detection of ASA in serum. Quality controls for the sperm antigen and anti-sperm antibody were tested to validate efficiency of the biochip. Meanwhile, serum ASA from male infertility was detected using the protein biochip platform in comparison with ELISA assay to provide evidences with the parameters in clinical usage. Furthermore, we investigated whether there were any differences in natural conception between ASA-positive cohort and ASA-negative cohort in one-year follow-up study.

## Materials and methods

### Biochip and proteins

Purified human sperm antigen and rabbit anti-human sperm IgG antibody and Mixed Antiglobulin Reaction (MAR) test kit were provided by Anhui Anke Biotechnology (Group) Co., Ltd. (Hefei, China) (http://www.ankebio.com/english/). The isolation of sperm membrane antigens and the production ASAs referred to previous publications, respectively [20,21]. Cy3-conjugated goat anti-rabbit IgG antibody was commercially purchased from Sangon Biotechnology Co. Ltd. (Shanghai, China). N-Hydroxysuccinimide (NHS)-modified biochips were supplied by Thermohybaid, Interactiva Division (Ulm, Germany). Facility for microarray scanner was LuxscanTM 10K-A (Capitalbio Co., Ltd. China).

### Patients and serum collection

**Figure 1:**
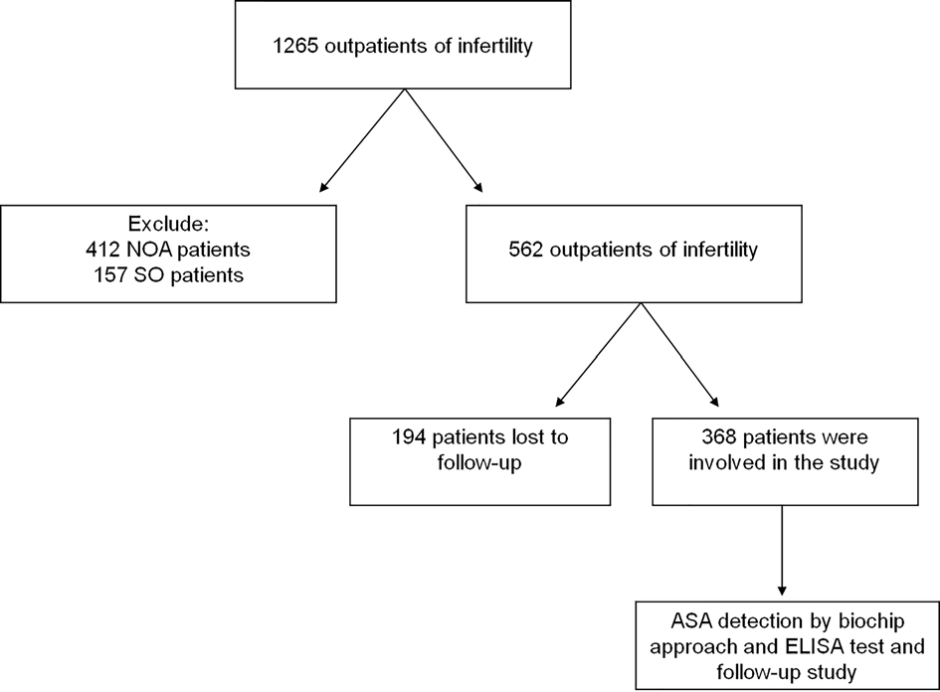
Scheme of ASA detections in a hospital-based study

The scheme for the study is depicted in Figure 1. After excluding the infertile reasons that might be caused by the females, a total of 1149 infertile male patients were recruited in the present study. Among which, 412 patients which were diagnosed as non-obstructive azoospermia (NOA) and 157 patients which were diagnosed as severe oligospermia (SO) were excluded for further testing of ASA. The diagnoses of SO, NOA and unexplained infertility were made by experienced doctors of obstetrician and gynaecologist. SO was defined as those males of sperm concentration <5 million/ml, and NOA was defined as azoospermia when no sperm was found after centrifugation at 3000 ***g*** for 15 min. If couples did not have conception more than 2 years, and meanwhile both of whom had no physiological and pathological abnormalities, we define the cohorts as unexplained infertility. After all, 368 of unexplained infertile males with completed 1-year follow-up investigation were recruited in the study. Semen parameters for the 368 unexplained infertile males for listed in Table 1. Sera were collected from the Reproductive and Genetic Hospital of XiangYa School of Medicine, Central South University, Hunan, China. The serum was stored at –80°C. A total of 116 controls were all fertility men who had fathered one or more healthy children. The mean age and standard derivation were 27.3 ± 5.27 years old for cases and 26.89 ± 4.71 years old for the controls. Informed consent was obtained from all individual participants included in the study. The present study was approved by the Clinical Research Ethics Committee of the Reproductive and Genetic Hospital of XiangYa School of Medicine.

**Table 1:** Semen parameters for 368 unexplained infertile males

Semen parameter	Mean ± SD
Volume (ml)	2.5 ± 0.9
pH	6.5 ± 0.7
Sperm concentration (million/ml)	122.2 ± 93.1
Progressive motility (%)	55.9 ± 19.0
Normal morphology (%)	40.2 ± 24.9

### Quality controls for the protein biochip

To optimize antigen immobilization on the protein biochip, biochips were rinsed with 0.01 M PBST buffer (phosphate-buffered saline, pH 7.4, Sigma; 0.1% Tween 20, v/v, Sigma) twice in room temperature (RT), and then dried with nitrogen. Human sperm antigen was diluted to a serial concentration of 100, 50, 25, 12.5, 6.25, 3.12, 1.56, 0.78, 0.39, 0.19 and 0.09 µg/ml, respectively, in 0.01 M PBST (pH 7.4) and 0.1% BSA (w/v, bovine serum albumin, Sigma). The various concentrations of human sperm antigen were immobilized onto the biochips. Each concentration of the antigen took 8 individual spots. Biochips were incubated at RT and in a humid chamber for 2 h. After rinsed with PBST (pH 7.4) three times for 2 min, and dried with nitrogen, biochips were incubated with 50 µg/ml of rabbit anti-human sperm IgG antibody in PBST-BSA buffer (pH 7.4) at RT and in the humid chamber for 1 h, 2.5 µg/ml (1:200 dilution) of Cy3 conjugated goat anti-rabbit IgG antibody in PBST-BSA buffer (pH 7.4) at RT and in the humid chamber and in a dark environment for 1 h, respectively. After rinsed again with PBST (pH 7.4) and dried, fluorescence signals on the biochips were scanned by the LuxscanTM 10K-A biochip scanner.

To test the minimal detectable amount of anti-human IgG antibody on the biochip platform, we immobilized 50 µg/ml of human sperm antigen in PBST buffer (pH 7.4) on the biochip for 2 h. Rabbit anti-human sperm IgG antibody was diluted to a serial concentration of 100, 50, 25, 12.5, 6.25, 3.12, 1.56, 0.78, 0.39, 0.19 and 0.09 µg/ml, respectively, in PBST-BSA buffer (pH 7.4). The antigen-immobilized biochips (50 µg/ml) were incubated with the serial concentrations of the rabbit anti-human sperm IgG antibody described above for 1 h, and with 2.5 µg/ml (1:200 dilution) Cy3 conjugated goat anti-rabbit IgG antibody in PBST-BSA buffer (pH 7.4) for 1 h, one by one. Finally, fluorescence signals on the biochips were scanned by the LuxscanTM 10K-A biochip scanner. Blank controls were interpreted by PBST-BSA signal following incubation of 2.5 µg/ml (1:200 dilution) Cy3 conjugated goat anti-rabbit IgG antibody in PBST-BSA buffer (pH 7.4).

### Serological detection of ASA by biochip

Human sperm antigen (50 µg/ml, PBST-BSA buffer, pH 7.4) probed biochip was prepared before serological immunoassay. Serum samples from 368 infertile and 116 healthy males were diluted 1:20 in PBST-BSA buffer (pH 7.4). We define healthy males as the man who had history of child bearing. Serum incubation was performed at RT for 1 h. The bindings of specific IgG antibody to sperm antigen on the biochips were detected by the Cy3-conjugated goat anti-human IgG antibody (2.5 μg/ml) in PBST-BSA buffer (pH 7.4) at RT for 1 h. Average fluorescence values of four known negative sera and PBST-BSA buffer were calculated as negative and blank controls, respectively. Fluorescence values three times higher than the average fluorescence value of the negative controls were evaluated to being positive.

### Detection of anti-sperm antibodies by ELISA and MAR

Binding IgG antibody to sperm antigen was measured by the standard ELISA, of which the method was according to a previous report [22]. In brief, polystyrene microtiter plates (Nunc, 96 wells, Denmark) were coated with 100 μl of sperm antigen (10 μg/ml, 0.05 M carbonate-bicarbonate buffer, pH 9.6, Sigma) at 4°C overnight. After blocked with 10% bovine serum albumin (w/v, Sigma), wells were incubated with 100 µl of the serum samples from the patients and the healthy donors at 37°C for 90 min. Samples to be evaluated were performed in duplicate. After washed, HRP conjugated goat anti-human IgG (1:500, Sigma) was incubated at 37°C for 60 min. Finally, wells washed and incubated with enzyme substrate TMB (tetramethylbenzidine, Sigma) and H_2_O_2_ (30%, v/v, Sigma) at RT for 15 min. After stopped the reaction with 2 M H_2_SO_4_ (50 μl/well), the developing color was quantified on an automatic microtiter plate reader (Bioreader, Model 550, Japan). Results were expressed as optical density units (OD) at 492 nm. Average OD values from 4 wells containing four known negative sera and 0.05 M carbonate–bicarbonate buffer 0.1% BSA were calculated as negative and blank controls, respectively. OD values of three times higher than the average OD value of the negative controls were evaluated to be positive. In another aspect, a total of 262 patients with unexplained infertile males were recruited for MAR test.

### Follow-up study for ASA-positive and ASA-negative cohort

A total of 368 infertile males were recruited in the follow-up to investigate whether natural pregnancy occurred during 1 year time after outpatient services. All patients did not accept any medical treatments or assisted reproductive technologies (ARTs) for promoting gestation. Meanwhile, all patients had definitely child-bearing willingness and attempted to have natural pregnancy methods.

### Statistical analysis

SPSS software (Version20.0) was applied in the present study. All enumeration data were provided as the mean ± standard deviation (mean ± SD) and analyzed with the *t* student test. Binary data were analyzed with the Chi-squared test. Correlation efficiency was calculated with Pearson’s correlation analysis. The *P* values reported in the study were based on a two-sided probability test with a significance level of *P* < 0.05.

## Results

### Optimizing detection of sperm antigen and anti-sperm antibody on biochips

**Figure 2:**
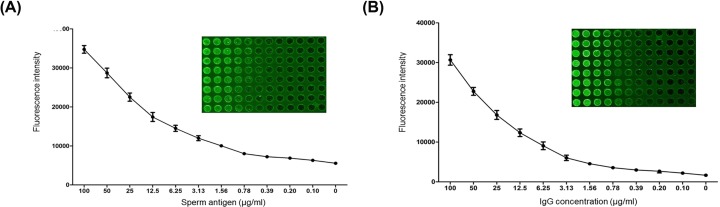
Immobilization of purified human sperm antigen and optimization of rabbit anti-human IgG antibody by biochip A duplicated dilution of sperm protein (0.09–100 µg/ml) on a N-hydroxysuccinimide (NHS)-modified biochip was tested (**A**). Similarly, optimized Cy3-based immunological assay was performed in a duplicated dilution of IgG antibody (0.09–100 µg/ml) (**B**).

Purified human sperm antigen and rabbit anti-human sperm IgG antibody were immobilized onto biochips at the serially diluted concentration of 100, 50, 25, 12.5, 6.25, 3.12, 1.56, 0.78, 0.39, 0.19 and 0.09 µg/ml in 0.01 M PBST-0.1% BSA (1 µl/spot), respectively. Three times of average fluorescent values over blank controls were evaluated to be positive. Figure 2A showed attenuation of the fluorescent intensity occurred as the concentration of immobilized human sperm antigen decreased on the biochip. The detectable limit of sperm antigen reached to the concentration of 0.78 µg/ml. Optimal fluorescent intensities to detect appeared on spots at concentrations ranging from 12.5 to 50 µg/ml. Figure 2B showed decay of the fluorescent intensity emerged as the concentration of immobilized rabbit anti-human sperm IgG antibody decreased on the biochip. Similarly, the visible detection limit of anti-sperm antibody was identified at the concentration of 0.78 µg/ml. Optimal fluorescent intensities were exhibited on spots at concentrations ranging from 12.5 to 50 µg/ml.

### Detection of serological anti-sperm antibody of infertile and fertile males by the biochip

**Figure 3:**
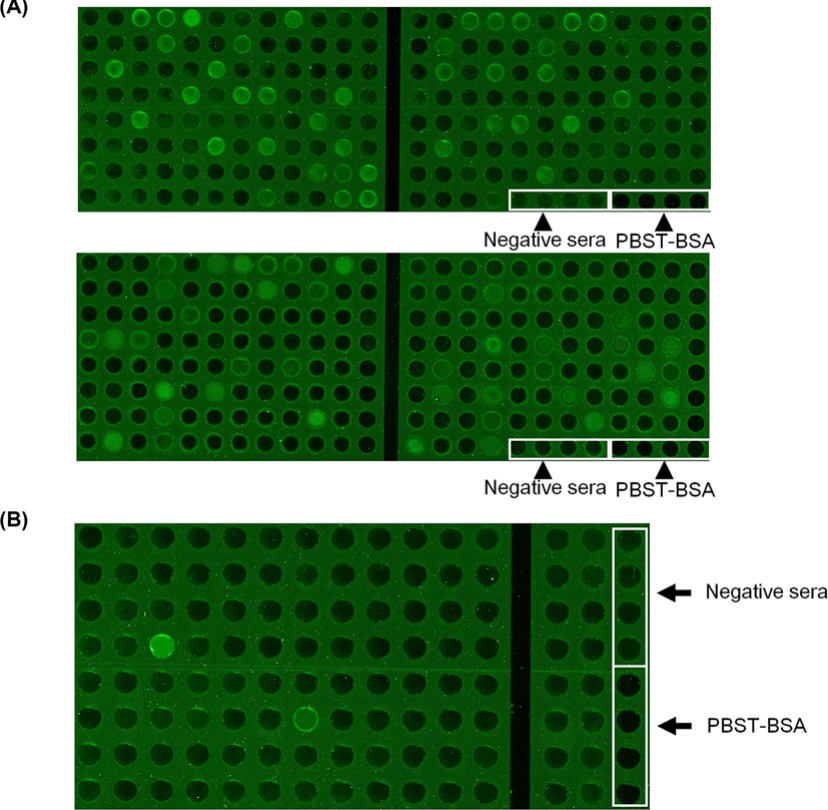
Biochip detection of serum ASA expression Screening of serum ASA in 368 infertile males (**A**) and 116 fertile males (**B**) by the biochips. Four known negative sera and PBST-BSA buffer were selected as negative and blank controls. The sera with the fluorescence intensities of three times higher than mean fluorescent value of the control sera were evaluated as being positive.

**Figure 4:**
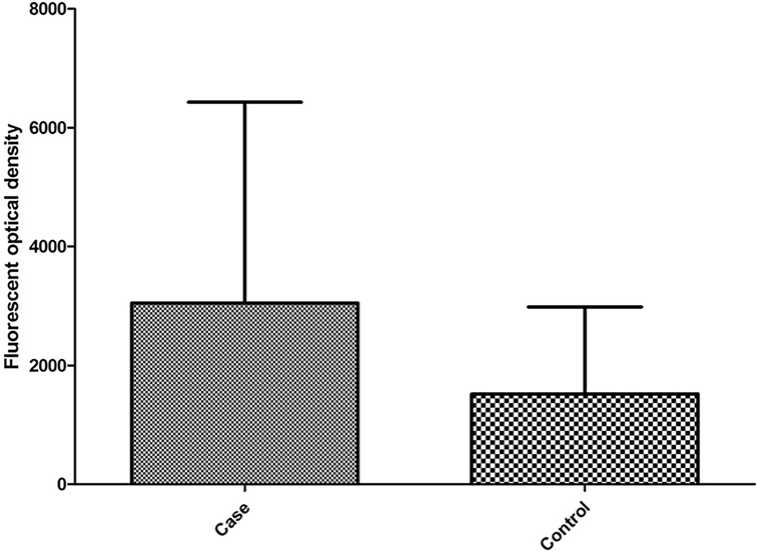
Distributions of quantitative fluorescent optical density for both cases and controls

**Figure 5:**
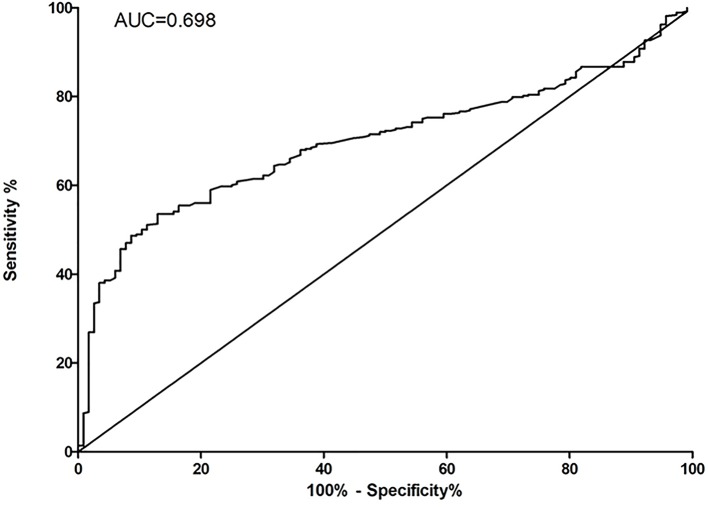
ROC analysis for serum ASA expression between infertile and fertile males

Serum ASA (1:20 diluted in PBST-BAS, pH 7.4) in 368 infertile (Figure 3A) and 116 healthy males (Figure 3B) was detected by using of the sperm antigen coated biochips (50 µg/ml). Positive rates of serum ASA were 20.92% (77/368) in the cases and 1.72% (2/116) in the controls, respectively. The total positive rate of serum ASA was 10.08% in the present study. The distribution of quantitative fluorescent optical density for both cases and controls were shown in Figure 4. ROC analysis between fertile males and infertile males for biochip-based technology was shown in Figure 5. The cut-off value for ASA-positivity was estimated to be above 1547 fluorescent optical density (FOD), and the sensitivity and specificity for diagnosis for infertile male from fertile male, by using ASA, was estimated to be 53.53% and 87.07%.

### Correlation analysis of serum ASA detection using biochip approach and ELISA test

**Figure 6:**
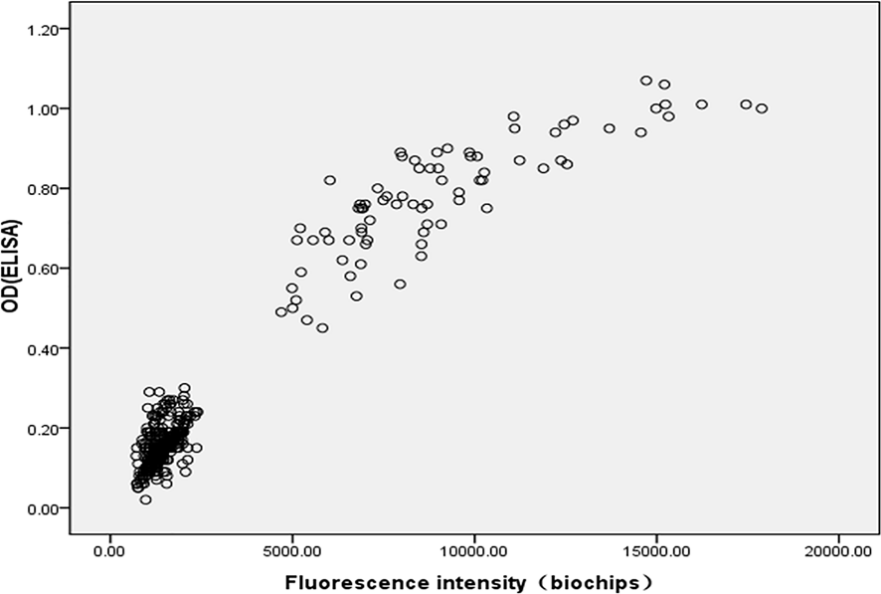
Correlation of biochip results versus ELISA

To verify the accuracy and detection biases of the biochip platform, the experiment was evaluated by an ASA based-specific ELISA approach. Figure 6 showed immunological reactions of ASA by the protein biochip that was completely proportional to that of ELISA. The correlation coefficient (*R*^2^) between the fluorescence values by the biochip and the OD values by ELISA assay at all the spots was 0.960, indicating that valuable application of the protein biochip and ELISA was compatible.

Also, we compared preponderance in ASA detection by the biochip, ELISA and MAR. We found that the positive rates of ASA expression were 20.92% by the biochip and 21.47% by ELISA assay. No significant differences were found between two groups (*P* > 0.05, Table 2). For another, in a relatively small testing cohort that included 262 patients, the positive rates of ASA expression were 20.62% by the biochip and 16.03% by MAR test, respectively. There were also no differences between the biochip and MAR test (*P* > 0.05, Table 3).

**Table 2:** Comparison of the biochip and ELISA assay in serum ASA detection

Approach	ASA-positive	ASA-negative	Positive rate	*P* value
Biochip	77	291	20.92%	0.857
ELISA	79	289	21.47%	

**Table 3:** Comparison of the biochip and MAR test in serum ASA detection

Approach	ASA-positive	ASA-negative	Positive rate	*P* value
Biochip	54	208	20.61%	0.175
MAR	42	220	16.03%	

### Analysis of natural pregnancy between ASA-positive and ASA-negative groups

In the 1-year follow-up of 368 infertile patients, natural pregnancy rate was much lower in ASA-positive patients (6.5%, 5/77) than that in ASA-negative patients (18.9%, 55/291). There was a significant difference between the two groups (*P* = 0.009, OR = 0.298, 95% CI 0.115–0.773) (Table 4).

**Table 4:** Natural conception rate in ASA-positive and ASA-negative infertile patients in 1-year follow-up investigation

		Natural conception			
		Yes	No	*P* value	OR (95% CI)
ASA	Positive	5	72	0.009	1 (Ref)
	Negative	55	236		0.298 (0.115-0.773)

## Discussion

There are few researches concerning the mechanisms of ASA toward infertility. Detecting anti-sperm antibody in serum and seminal plasma is routinely carried out for the diagnosis and evaluation of infertility of couples in most reproductive centers in China.

In previous studies, sperm has been considered as an antigenic for the human immune system, and ASA was once demonstrated to be a influential factor for the natural conception rate and some treatments toward ASA could increase the pregnancy in parts of subfertile individuals [23–25]. Blood–testis barrier detaches sperms and its precursor cells from human immune system. Higher ASA response observed in the male’s blood would be inclined to decrease the rate of couple’s pregnancy [26]. Increasing expression of serum ASA seemed proportional to that in the seminal plasma. However, the exact effects of ASA in reproduction and ARTs applications remained controversial [27]. As the complexity of sperm antigens and, most importantly, the development of ARTs, females could get pregnant, bypassed the effects of ASAs by using modern ARTs. We inferred that why more and more research spend less time for the ASA. However, according to some previous researches and the follow-up results in our study, we also realized that ASA did have some negative effects in the natural conceptions. In the present study, we aimed at presenting a biochip-based assay for fast detection of ASA in the serum of Chinese infertile and healthy males. The results showed that the positive rates of ASA in the infertile and healthy males were 20.92% and 1.72%, respectively. There was a significant difference between infertile males with unknown causes and healthy males. However, it was reported that ASA did not affect the process of pregnancy for those who were implemented by the assisted-reproductive technologies, such as *in vitro* fertilization (IVF) and intracytoplasmic sperm injection (ICSI) [27–29]; therefore, more researches were focused on whether the ASA exerted the external environment of germ cells and affected the process of sperm-oocyte binding. Although reasons of how ASA production was induced and how ASA functioned toward infertility remained yet unclear, ASA might have a negative impact on male fertility through the following mechanisms: (i) ASA might interfere with the motility and penetration of sperm through cervical mucus and disturb the sperm-oocyte binding; (ii) ASA could adversely affect the acrosomal reaction through interference of processes of capacitation; (iii) ASA might make spermatozoa agglutinate; and (iv) ASA could indirectly mediate release of cytokines that could impair the function of sperms [13,30–34].

On the other hand, we conducted the follow-up screening for the natural pregnancy rate in the recruited subjects in order to confirm the correlation between ASA presence in male’s serum and its negative effects in pregnancy. The results showed that the ASA expression in serum was significantly correlated with natural pregnant rate. ASA-positive male infertile patients had a low natural pregnancy rate in comparison with ASA-negative patients, also demonstrating that the potential function of ASA in naturally reproductive processes. That probably implied the importance of ASA detection previous to any ARTs application for the infertile males with unknown etiology. However, relatively low-throughput for the traditional approaches in ASA detection might impede the wide applications in a large cohort screening. This biochip-based approach might provide a simple and potential tool for the clinical diagnosis and the mechanism study of the entity.

Although MAR test was recommended by World Health Organization (WHO), ELISA is one of the conventional assays to detect serum ASA in hospitals of China. In the present study, we found that the biochip-based assay showed a significant consistency to ELISA, the coefficient of determination from standard calibration curves of ASA was measured at *R*^2^ = 0.960, suggesting this protein biochip assay was capable of detection for the specific anti-sperm antibody and shared a similar outcome with ELISA method. Additionally, we also detected seminal ASA expression by MAR test. Although there were no significant differences between results of MAR test and biochip approach, results by using MAR test had lower positive rate than those of biochip approach and ELISA test.

However, it should be indicated that the biochip-based approach like any other protein assays had its shortages in detection of the serum ASA. Two forms of ASA occurred in infertility: free ASA and combined ASA. ASA that led to the infertility often was the one that combined to the sperms. In addition, investigation of the ASA combined on the surface of the activated sperm would probably be more valuable rather than that binding on the surface of the inactive sperm. As there were a variety of sperm-related antigens that could cause the expression of ASA in both semen and serum, MAR test, which was a gold standard for ASA detection in semen, detected the combined antibodies on the surface of the motile sperms, while the serum ASA that ELISA and biochip tests detected might be from both free and combined ASA. Therefore, the positive rate of ASA by ELISA and biochip tests would be higher than by MAR test. Although ASA expression in the serum was inclined to be consistent with that in the seminal plasma, it seemed yet difficult to deduce influence of the free ASA and/or the combined ASA in the seminal plasma by detecting serum ASA. Interpretion of clinical significance of the serum combined ASA by the biochip-based assay would be an new challenge to perform reproductive-associated antibody screening and to make clinical decision.

## Conclusion

We provided a novel biochip-based approach for detection of serum ASA expression. The biochip-based detection for serum ASA showed a significant consistency with ELISA. Meanwhile, we found serum ASA expression was significantly correlated with natural pregnancy rate. This platform would be both feasible for screening the serum ASA expression in a large cohort of infertility and further investigation of ASA function.

## Abbreviations

ASA: anti-sperm antibody
ELISA: enzyme-linked immunosorbent assay
FOD: fluorescent optical density
IBT: immune beads test
ICSI: intracytoplasmic sperm injection
IVF: *in vitro* fertilization
MAR: mixed anti-globulin reaction test
NHS: N-hydroxysuccinimide
NOA: non-obstructive azoospermia
ROS: reactive oxygen species
SO: severe oligospermia
